# Progress on the Application of Polymers of Intrinsic Microporosity for the Adsorption of Organic Contaminants

**DOI:** 10.1002/open.202500430

**Published:** 2025-11-07

**Authors:** Martins O. Omorogie

**Affiliations:** ^1^ Chair of Urban Water Systems Engineering School of Engineering and Design Technical University of Munich Am Coulombwall 3 Garching Germany; ^2^ Department of Chemical Sciences Faculty of Natural Sciences Redeemer's University P.M.B. 230 Ede Nigeria; ^3^ African Centre of Excellence for Water and Environmental Research (ACEWATER) Redeemer's University P.M.B. 230 Ede Nigeria

**Keywords:** adsorption, organic dyes, pharmaceuticals, polymers of intrinsic microporosity‐1/polymers of intrinsic microporosity, polymers of intrinsic microporosity

## Abstract

This review reports the progress on the utilizationof polymers of intrinsic microporosity (PIMs) for the adsorption of pharmaceuticals (PCs) and organic dyes. PIMs are exceptional porous organic polymers that possess copious contortion sites and rigid fused‐ring structures induced by spirocentric molecules (two cyclic rings sharing one tetrahedral carbon). The availability of these contortion sites inhibits bond flexibility, bond rotation, and structural relaxation of PIMs in their solid state. This has led to the intrinsic microporosity, high Brunauer–Emmett‐Teller and Barrett–Joyner‐Halenda surface areas, pore radii, pore volumes, high permeability, high diffusivity, high selectivity, and high thermal stability. PIMs comprise a cascade of girthy ladder‐like building blocks connected to the spirocentre as a result of inflexible backbone stereochemistry. Research progress has shown from a thorough literature survey that the adsorptive properties of PIMs and their functionalized analogs have not been extensively explored for the removal of PCs and organic dyes in contaminated water. To date, there exists scanty literature on the adsorption of PCs in contaminated water. In prospect, research efforts have to be intensified so as to establish vast applications of PIMs for the treatment of water contaminated with PCs and organic dyes.

## Graphical abstract

The pictorial illustration of the applications of polymers of intrinsic microporosity extraordinary porous organic polymers with high surface areas, pore radii, pore volumes, high permeability, high diffusivity, high selectivity, and high thermal stability, as suitable filters or purifiers for water contaminated with pharmaceutical and organic dyes.

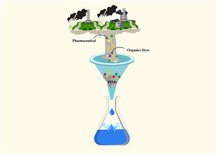

## 1 Introduction

**FIGURE 1:**
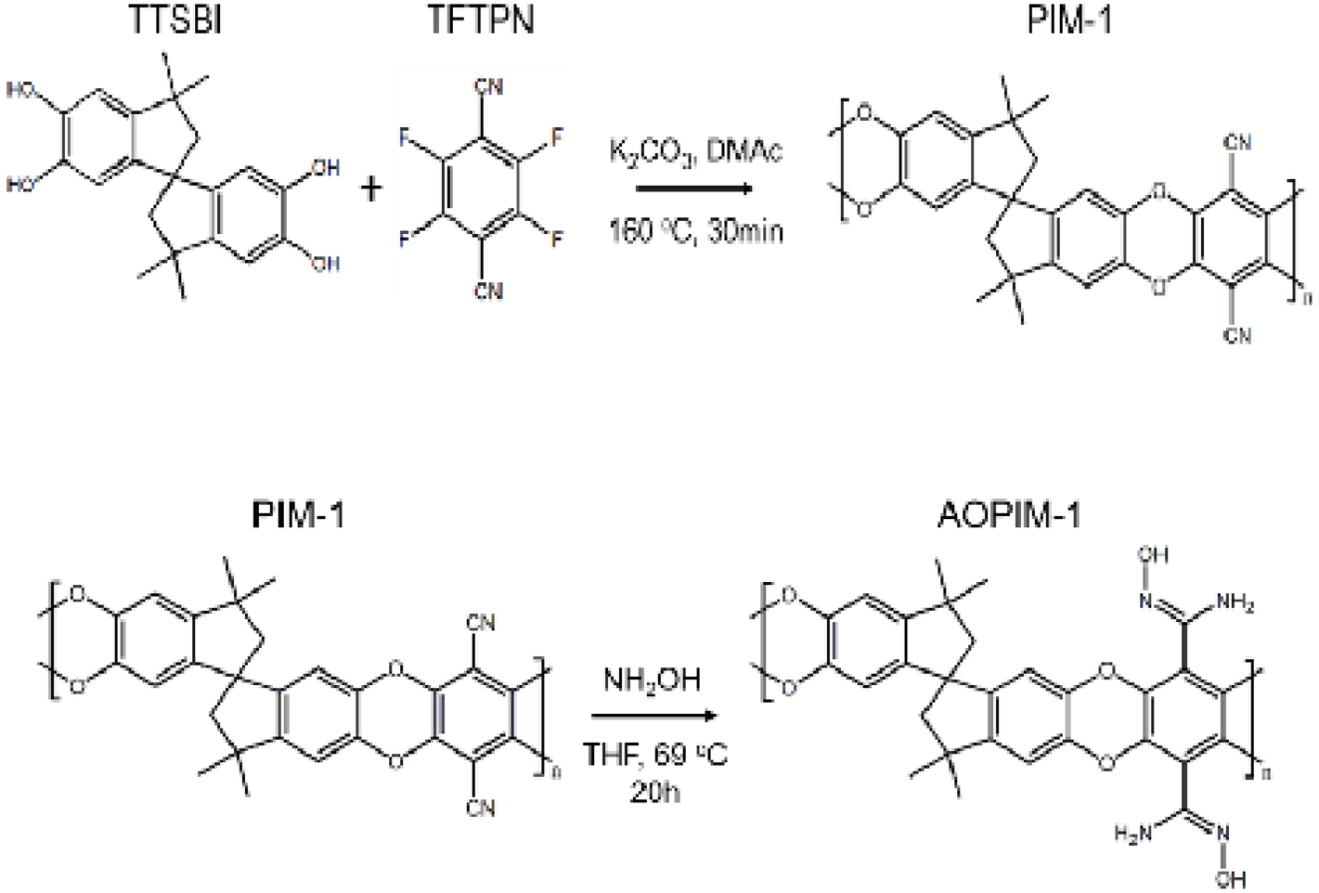
The synthesis of PIM‐1 and AO‐PIM‐1 [27].

**FIGURE 2:**
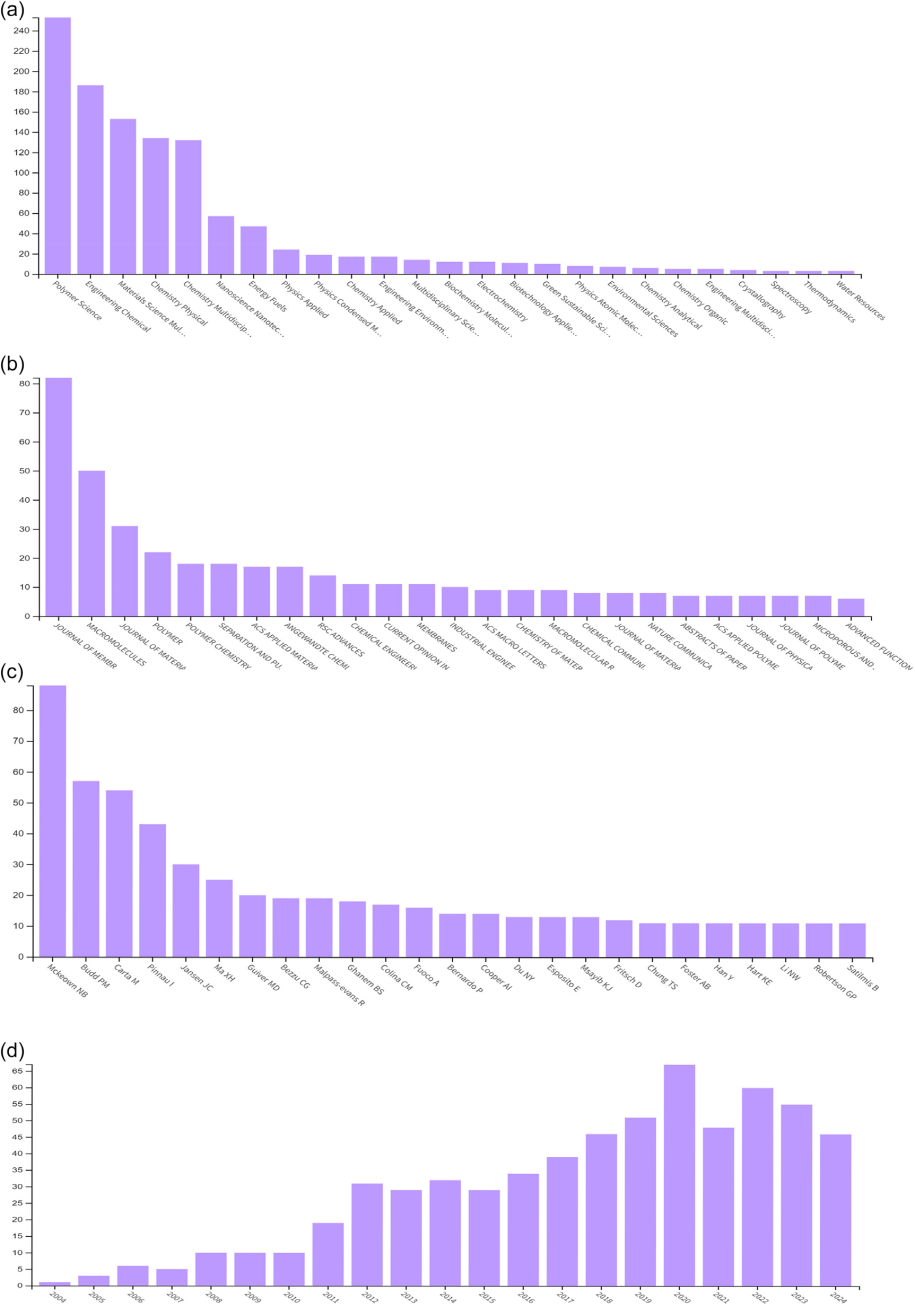
Number of papers in PIMs that were published by (a) various fields of science, (b) various highly reputable Journals, (c) various Authors, and (d) years. Accessed on the 9^th^ January, 2025, from Clarivate Analytics Web of Science.

There has been swift progress in the synthesis of microporous network polymers for various environmental applications in recent years [1]. There has been a significant development in the pathways of synthesis as regards various polymer networks that have led to the nucleation and formation of amorphous and crystalline microstructures. The plausible incorporation of various moieties in concerted succession is the hallmark of the functionalization of polymers, engineered to specific applications [1, 2]. In recent years, there has been a tremendous advancement in the development and environmental applications of structured nanoporous polymer networks [3, 4–6]. These applications have successfully covered a wide array of separations in the form of air purification, water/wastewater treatment and gas separation; storages in the form of gas storage and CO_2_ capture, catalysis in the form of reduction of toxic gases such as CO_2_ and CO reduction, NO_*x*_ reduction, energy storage in the form of capacitors and super‐capacitors, etc. [1, 2, 7, 8, 11, 12–13]. Polymers of intrinsic microporosity (PIMs) are structured nanoporous materials that have drawn the vast attention of Researchers/Scientists in recent years due to their excellent tunability, surface engineering, well‐defined surface chemistry, high porosity, and suitability as molecular sieves for various environmental applications. PIMs are polymer‐based organic microporous compounds that are special polymers with bulky and rigid contortion sites in the polymer backbone. Their microscale porosities are formed from the rigidly curved monomers that comprise the sp^3^ carbon atom responsible for the ineffective filling of spots. These spots metamorphose into open volume and accessible pores among the polymer chains after the removal of solvents [2, 14, 15]. According to the International Union of Pure and Applied Chemistry, microporous materials are materials whose particles have a pore diameter of < 2 nm [16]. PIMs possess very high interconnectivity in their microporous networks, making them to have structural variability and an extraordinary high surface area [2, 17]. Materials of high microporosity are solids that possess well‐structured and large surface areas, ranging from 250 to 2500 m^2^ g^−1^ by virtue of their gas adsorption. Zeolites, activated carbons, and other molecular sieves are the conventionally known materials of high microporosity over the years. These conventional materials of high microporosity mainly possess unique homogeneity and chemical structure that are engineered toward multifarious applications that do not require heterogeneity and chemo‐selectivity. The quest for the demand and applications of multifunctional materials of high microporosity for air purification, water/wastewater treatment, gas separation, gas storage, carbon dioxide capture, catalysis, and energy storage, which require heterogeneity and chemo‐selectivity, brought about the synthesis of network and hyper‐crosslinked polymers (HCPs) with intrinsic microporosity and high surface areas of 800–1300 m^2^ g^−1^. These PIMs were polystyrene and polyarylcarbinol. Thereafter, the concept of introducing robust microporosity into polymers arose from intense research work that focused on the incorporation of extended aromatic moieties, with the target of the mimicry of graphene components of activated carbons, so as to produce a rigid polymer network. Further discovery led to the synthesis of phthalocyanine network polymers (PNPs). These PNPs agglomerated into nonporous aromatic components that revealed strong noncovalent columnar stacks (essentially *π–* *π* stacking), resulting in nonporous solids. More research led to the synthesis of phthalocyanine subunits with nonlinear, tough, and rigid linking moieties (5,5′, 6,6′‐tetrahydroxy‐3,3,3′, 3′‐tetramethyl‐1,1′‐spirobisindane) among the subunits. This attempt succeeded in the infusion of irregular space packing that hampered increased microporosity and structural relaxation. Further attempt to create regular space packing and highly microporous PNPs from the cyclo‐tetramerization of bis(phthalonitrile) precursor on metal ion template. The bis(phthalonitrile) precursor was synthesized from a double aromatic nucleophilic substitution reaction of 5,5′, 6,6′‐tetrahydroxy‐3,3,3′, 3′‐tetramethyl‐1,1′‐spirobisindane and 4,5‐dichlorophthalonitrile to form dioxane and network polymers with spirocyclic crosslinks. These network polymers successfully gave rise to unique amorphous PIMs that were void of the close packing and rigidity of Phthalocyanine components, giving rise to amorphous microporous polymers with Brunauer–Emmett‐Teller (BET) surface areas of *ca.* 1000 m^2^ g^−1^ at 77 K after nitrogen gas physisorptometry [9, 14, 18, 19, 20, 21, 22, 23, 24, 25–26]. Figure 1 shows the various reaction conditions for the synthesis of PIMs. Figure 2a–d revealed that PIMs have found applications in various fields of science in the last two decades, most especially as membranes and adsorbents which served as molecular sieves for water/wastewater treatment, gas separation, gas storage, carbon dioxide capture, catalysis, and energy storage, etc. Herein, this paper is a comprehensive review of the recent advances in the applications of PIMs for the removal of pharmaceuticals (PCs) and organic dyes in water.

## 2 Synthesis of PIMs

The synthesis of PIMs began in 2001 with the formation of a polymeric network from spirobisindane and phthalocyanine [14, 15]. This attempt led to the synthesis of TTSBI and tetrakis‐meso (pentafluoro phenylporphyrin), which were utilized as precursors for the synthesis of PIMs by Budd et al. [28]. The synthesized PIMs from the catalytic oxidation of these precursors appeared as yellow amorphous fluorescent powder with high molecular weight and surface area. The high molecular weight and surface area of PIMs are attributable to the nonflexibility of spirobisindane and benzodioxin. Also, the solid‐state PIMs formed displayed nano‐dimension pores with contorted structure and free volume [10, 29–32]. The availability of contortion and nonflexibility in the structure of PIMs decreases within the intermolecular cohesive interactions and, in turn, increases their solvation activity. Conversely, for some PIMs, when the reduction in contortion and nonflexibility of their structure decreases within the intermolecular cohesive interactions, it would lead to decreases in their solvation activity. Conversely, the introduction of flexibility and noncontortion into the structure of PIMs increases their intermolecular cohesive interactions and solvation activity. Generally, it has been revealed that PIMs with aromatic monomers with higher rigidity possess a higher BET surface area. However, some PIMs synthesized with flexible constituents, such as tetrahydro‐naphthalene have lower permeability and microporosity when compared with those from spirobisindane and dibenzodioxin constituents [33, 35, 36–37].

**FIGURE 3:**
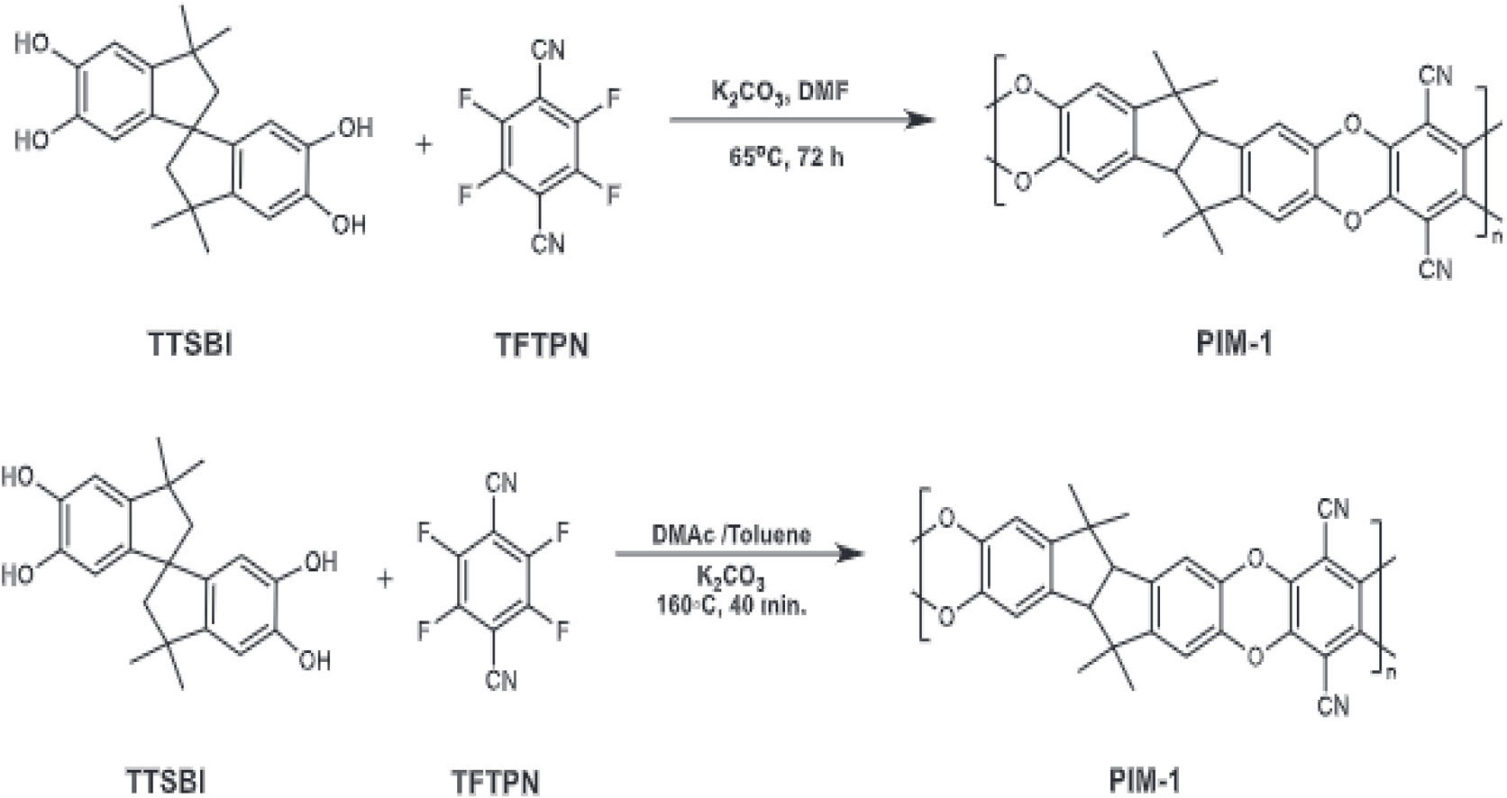
The synthesis of PIM‐1 at 65°C and 160°C (Note: 5,5′,6,6′‐tetrahydroxy‐3,3,3′,3′‐tetramethyl‐1,1′‐spirobisindane (TTSBI) and 2,3,5,6‐tetrafluoroterephthalonitrile (TFTPN)) [39].

**FIGURE 4:**
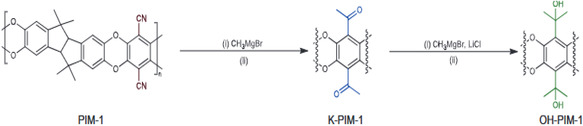
The synthesis of ketone‐PIM‐1 (K‐PIM‐1) and alcohol‐PIM‐1 (OH‐PIM‐1) from PIM‐1 [39].

The molecular chains of PIMs show the formation of three plausible conformations, namely ladder, linear, and network. The ladder molecular chain conformation is commonly the plausible chain conformation for PIMs. The polymerization of dibenzodioxin to give archetypal PIM‐1 is a plausible ladder molecular chain conformation. Also, the network molecular chain conformation is found in PIMs with average functionality >2. This implies that the branches of their polymers increase multidimensionally and form a convoluted network structure, *e.g*., phthalocyanine‐PIMs [14] and triptycene‐PIMs [38] (Figures 3 and 4).

### 2.1 Plausible Molecular Chains of PIMs

#### 2.1.1 Ladder Conformation via Dibenzodioxane

Aromatic nucleophilic substitution mechanism was used for the synthesis of soluble PIMs by polymerization of bis catechol‐containing monomer and tetrahalide to give dibenzodioxin‐linked PIMs. This polymerization had been successfully used for the synthesis of high average molecular fused ring ladder‐type PIMs and network PIMs ab initio. In pragmatic terms, a nucleophilic substituted halogen monomer that acts as an electron‐withdrawing substituent is a cogent requisite for this reaction. Ladder‐type PIMs were synthesized by low and high‐temperature techniques. The synthesis of PIM‐1 at low temperature via equimolar ratio of monomers with dry anhydrous K_2_CO_3_ under inert atmosphere at 65°C for 75 h [31]. Considering the dibenzodioxin polymerization, it was effective for the synthesis of PIM‐1 and new PIMs for membrane applications. Low‐temperature techniques have been found to be effective for the synthesis of network and linear polymers. The high temperature technique involves the rapid stirring of the precursors in dimethylacetamide with toluene at 160°C, which results in a high average molecular weight polymer [30, 40, 41]. This is the best technique for the synthesis of PIMs due to its short‐time polymerization reaction (PIMs were synthesized within 8 min), improved mechanical properties, and the production of meager amounts of microcyclic, oligomeric, and crosslinked fractions [42]. This technique is simplistic, impacts permeability, high BET surface area, and high solubility of PIMs. However, some Researchers used low temperature techniques to synthesize spiro‐benzodioxane‐based PIMs, various cyclic ladder oligomers and polymers via dibenzoxane reaction based on trimethylsilyl‐derived bis‐catechol, facile and benign high molecular weight ladder‐type PIMs [30, 43, 44], bis(phenazyl) based PIMs, PIM‐7, cardo‐PIM‐1, spirobifluorenes‐based PIMs [34], methyl, benzo, and t‐butyl‐based PIM‐2 [45], hexaphenyl benzene‐based PIMs, [46, 47] and other PIMs [48].

### 2.2 Tröger Base

Tröger base (TB) is known as 2,8‐dimethyl‐6H, 12H‐5,11‐methanodibenzo[b, f [1, 5] diazocine. It is synthesized by an electrophilic aromatic substitution reaction between proxy formaldehyde/formaldehyde and aromatic amine. TB is formed in a single high‐yield reaction protocol as a six‐covalent‐bridged bicyclic amine [49]. TB possesses special structural features that comprise contortion sites and high rigidity. These special features have attracted the attention of Researchers/Scientists to consider the applications of TB in supramolecular chemistry and molecular recognition [50, 51]. Also, TB is a stronger base than most amines due to its rigid bicyclic constraints that decrease the degree of conjugation among the aromatic rings and lone pairs of nitrogen [52]. Multifarious scientific methods have been successfully applied for the synthesis of TB under various conditions. Carta [53] synthesized TB‐based PIMs from dimethoxymethane and bifunctional aniline monomer, which served as a CH_2_ supplier in CF_3_COOH. Also, in another research, Carta et al. [54] unveiled the polymerization of TB in THF using bridged bicyclic 2,6(7)‐diamino‐9,10‐dimethylethano‐anthracene monomer. The resultant polymer demonstrated high solubility in CHCl_3_ with a high surface area and revealed high gas separation properties for some gases. Carta and his Colleagues [55] further made progress in this research by synthesis of trypticynene‐based TB intrinsic polymers. Rose et al. [56] synthesized a new PIM from benzotriptycene using the polymerization of TB via monomer addition into dimethoxymethane, CF_3_COOH. The TB obtained possessed high solubility in CHCl_3_, high microporosity, and excellent thermal stability. Sydlik et al. [57] synthesized PIM from 2,6‐diaminobenzotriptycene monomer and obtained PIM with an equal mixture of diamino regio‐isomers. In recent research findings, Carta and his research group synthesized 14 new PIMs from TB comprising monomers (tri(amino)triptycene, di(amino) ethanoanthracene, tri(amino) phenylbenzene (TAPB), and TAPB extended analog with structural diversity that would enable the tunability of pore size, various lengths of aromatic moieties, and diverse geometries [58]. These PIMs were highly microporous due to the availability of their contortion sites and rigidity [38, 48, 59, 60]. They synthesized PIMs with a high BET surface area (*S*_*BET*_) of 1027 m^2^.g^−1^ and an average pore diameter ( $\overset{―}{D_{p}}$) of < 2 nm from a trifluoroacetic acid‐mediated reaction between dimethoxymethane and 2,6(7)‐diamino‐9,10‐dimethyl‐ethanoanthracene or bis(4‐amino‐3‐methylphenyl)‐methane [61, 62, 63, 64, 65, 66, 67, 68, 69, 70, 71, 72, 73, 74, 75, 76, 77, 78, 79, 80, 81, 82, 83, 84, 85, 86–87]. Another technique for synthesizing PIMs from TB is the polycondensation between dianhydride/diamine and TB‐containing diamino. Typically, PIM is synthesized from 2‐methyl, 1,3‐benzenediamine (DAT) and 2,5‐dimethyl‐1,4‐phenylenediamine (DPD) [88, 89].

### 2.3 Catalytic Norbornene‐Arene Annulation (CANAL) Ladder PIMs and Phenazine

**FIGURE 5:**
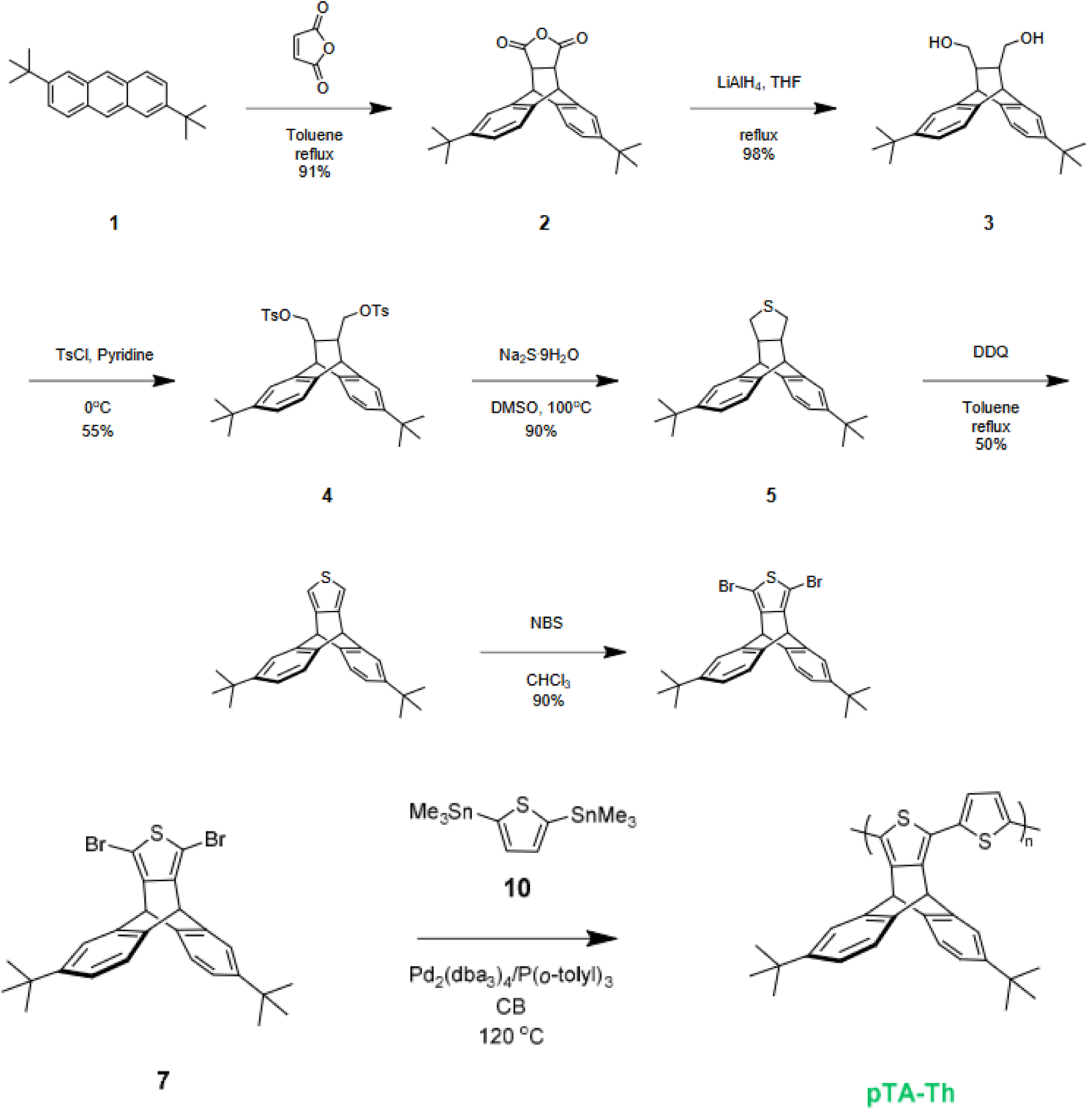
Reaction scheme for the synthesis of thiophene polymer (pTA‐Th) [103].

Phenazine‐rich ladders PIMs comprise successive rigid phenazine units with a building block in PIM‐1 other than dioxane networks. Fascinatingly, several PIMs reaction utilizes AA‐BB type self–polymerization interaction does not require adherent control of the starting monomers stoichiometric ratios during the synthesis of PIMs [59, 90, 91]. The PIMs formed show high molecular weight and solubility in organic solvents and high thermal stability. Ghanem et al. [90] synthesized ladder PIMs with effective A‐B type synthesis comprising a two‐bridge‐head at 9,10‐branched diisopropyl triptycene and phenazine having a highly fused rigid structure. Recently, CANAL ladder PIMs with antiaromaticity from their unique four‐membered rings that led to special materials having incredible annulation chemistry were synthesized by aryl bromides and norbornenes by some Researchers [92, 93, 94, 95, 96, 97, 98, 99, 100, 101–102] (Figure 5).

### 2.4 Post‐Modification of PIMs

Post‐modification protocols resulting from chemical modification(s) have led to a decrease in the structural integrity, porosity, solubility, and even stability of PIMs [104]. The engineered target in the synthesis of PIMs is to generate a facile technique that helps to greatly improve their free volume, microporosity, and gas permeability, extraordinary chain rigidity, and excellent solubility. Saliently, facile techniques are developed in line with post‐modification protocol(s) to suit the specific applicability of PIMs. PIMs are fabricated into efficient molecular sieves via step‐growth polymerization due to their rapid and selective molecular transport [105, 106]. These post‐modification protocols are;

#### 2.4.1 Nonsolvent Approach

This approach is needed and cogent for the preservation of the microporosity and surface area of PIMs. It endeavors to impede the transformation and conversion of functional moieties, such as amines, imines, hydrazones, imidazole, alcohols, ketones, etc. In this way, these functional moieties are kept intact by step using volatilization and counterintuitive nonsolvent method. It is interesting to note that this approach of post‐modification has increased the microporosity of PIM‐1 and accessibility of reactive functionalities of PIMs [104].

#### 2.4.2 Crosslinking Approach

This approach is viable for the synthesis of molecular sieves with high selectivity and exceptional gas permeability. The degree of crosslinking depends on catalyst specifics, reaction time, and reaction temperature and gives rise to PIMs that are excellent molecular sieves for gas transport. An increase in the degree of crosslinking gives rise to PIMs that are more thermally stable. Thermal crosslinking of PIMs increased their thermal stability due to the formation of stable planar triazine rings, which results in the formation of contracted cavities, a decrease in the free volume and interchain distance, and an increase in permeability and selectivity. Surge in inefficient chain packing and restrictions from conformational freedom are the viable reasons for these PIMs enhancements [100, 107].

Also, it is good to note that crosslinking constitutes a vital procedure to decrease gas‐induced plasticity and swelling in PIMs, thereby enhancing their tension/rigidity and molecular sieving behavior [108, 109]. Crosslinking can be achieved by some pathways, which are (a) ultraviolet (UV) radiation‐induced crosslinking, (b) azide‐aided crosslinking, (c) multivalent ionic crosslinking, (d) decarboxylation‐induced crosslinking, and (e) thermally induced self‐crosslinking. Khan et al. revealed that azide‐aided crosslinking led to high CO_2_ plasticity resistance and reduced permeability due to increased efficient polymer chain packing [110]. Li et al. revealed that decarboxylation and thermally induced crosslinking led to a decrease in permeability, high selectivity, and CO_2_ plasticity resistance [111]. Dong and Lee [112] revealed that UV‐induced crosslinking of PIM‐1 decreased microporosity, free volume, and permeability, increased selectivity, and altered spiro‐structural components. Zhao et al. [113]. and Rukmani et al. [114]. revealed that multivalent ionic crosslinking increased the size‐selectivity and rigidity, eradicated swelling, and increased CO_2_ plasticity resistance. Zhang et al. revealed that the crosslinking of triptycene‐based Tröger's base from 2,6‐diaminotriptycene diamines and 2,6‐diaminotriptycene‐14‐carboxylic acid led to the enhancement of gas transport, assuagement of the physical aging, and CO_2_ plasticity resistance. It was observed that the d‐spacing in these copolymers reduced as the carboxyl group concentration increased (resulting from the induction of strong hydrogen bonds, which stiffen adjacent polymer chains that led to increased selectivity and molecular sieving dynamics, and deterioration of gas permeability [115].

#### 2.4.3 Thermal Treatment Approach

This approach could be achieved via the thermal rearrangement of films comprising ortho‐localized moieties, such as hydroxyl gives PIMs having a benzoxazole moiety and thermal cyclodehydration. PIMs that are thermally treated have satisfactory selectivity, favorable permeability, and excellent plasticity resistance. To set a criterion/benchmark for free volume and porosity distribution is a functional dynamic to enhance diffusion pathways and interconnectivity among pores and large free volume content [116, 117, 118, 119, 120, 121, 122, 123, 124–125]. The addition of OH– into PIMs at the ortho‐position near the imide moieties supports the *α*‐type thermal conversion. The pendant moiety or contortion site can be in a diamine monomer or dianhydride. Previous research revealed that contorted dianhydrides resulted in improved gas permeability in comparison with their diamines’ analogs [112]. Thermal rearrangement occurred for dianhydride‐based PIMs having spiro centers, which positively impacts on their gas permeability [126]. The tunability of free volume and pore size distribution is an efficient means for increment in diffusion pathways. It is good to note that the thermal cyclodehydration and rearrangement are paramount in the formation of distinct micropores that are interconnected with high free volume. The possibility of an increment in the high free volume component is evident from the metamorphosis of packed PIMs into aromatic stiff‐rod‐like PIMs [125, 127].

#### 2.4.4 Functionalization (Moietization Treatment Approach

The introduction of moieties into PIM is being explored by various Researchers in recent times. This gives an opportunity to improve the physicochemical characteristics of PIM according to engineered or tuned applications. The versatility of the nitrile moiety in PIM‐1 makes it flexible for its conversion to various moieties, such as amine, amidoxime, thioamide, tetrazole, and as a carboxylic acid in one or more reaction steps. These new moieties that are formed greatly influence the fractional free volume, gas permeability, polymer–polymer, and gas‐polymer interactivities of PIMs. Carboxylate‐functionalized PIM, which comprises *ca*. 92 mol % of carboxylic acid moieties by a 360 h alkaline hydrolysis process. The COOH‐ PIM‐1 obtained had a lower interchain distance in comparison with the PIM‐1 [128]. Tetrazole and methyl tetrazole functionalized PIM‐1 are gas‐philic, highly soluble, and selective [112, 129]. Polar amidoxime functionalized (PAF) PIM‐1 has high microporosity and inflexibility. The PAF‐PIM‐1 has enhanced diffusivity, selectivity, permeability, and intermolecular hydrogen bonding in its network. On exposure to a gaseous system, it decreased matrix dilation and unfavorable [130]. Adamantine was grafted onto PAF‐PIM‐1 by a substitution reaction of acyl chloride [131]. Also, carboxylated PIM was synthesized by in situ hydrolysis of the nitrile moieties of PIM‐1 films. For crosslinking, grafting, and other modifications, carboxylic acid moieties are the preferential sites where they could take place. It is good to note here that carboxylation enhances the solubility of PIM‐1 and its modified analogs in polar aprotic solvents [132, 133–134]. Also, carboxylic dianhydrides based on TB and 3,3′‐dimethylnaphthidine were used to synthesize carboxylic PIMs, which possess high microporosity, selectivity, and permeability [135]. Borane complexes and dimethyl sulfide were used to synthesize amino‐functionalized PIM‐1 (AFP) with multidegree conversions. The AFP‐1 possesses reduced free volume, permeability and diffusivity when interacting with gases [136, 137]. On the other hand, the reaction of PIM‐1 with ethanolamine and diethanolamine gave AFP‐1 with increased free volume, permeability, and diffusivity when interacting with gases [138]. The AFP‐1 synthesized by in situ crosslinking and solid‐state deprotection has high selectivity and permeability [139].

In recent times, pH‐sensitive amidoxime‐modified PIM (PsPIM) was synthesized by the nonsolvent‐induced phase separation technique with ethanol as the nonsolvent. This PsPIM demonstrated high microporosity and selectivity for cations/anions at varied pH conditions [140]. In another research finding, hydrophilic metal–organic frameworks (MOFs) were used as active substrates with moieties that combined with surface post‐imprinting modification (sPIM). The surface engineering of sPIM led to the formation of hydrophilic dual‐receptor post‐imprinting modified polymers (dual MOFs‐rMIPs), which had high selectivity and permeability for water‐soluble 5′‐adenosine monophosphate (AMP). The esterified ammonolytic covalent interactions of dual MOFs‐rMIPs were utilized for in situ synthesis of imprinted polymers on the hydrophilic Zr‐MOFs nanosheets surface involving Zr^4+^/PO_4_^3–^ and the sequential assembly of the template AMP [141]. Also, porous hydrogels of amidoxime modified PIM‐1 (aPIM‐1) were synthesized by introducing into sodium alginate (SA) solution and adding CaCl_2_ into the mixture at low temperature for crosslinking. This synthesis yielded highly microporous SA/aPIM‐1 hydrogels with high selectivity for Rhodamine B (RhB) [142]. The facile synthesis of aldehyde‐functionalized PIM‐1 (PIM‐CHO) from PIM‐1 films microstructure by converting the nitrile groups of PIM‐1 into aldehyde groups at room temperature. The PIM‐CHO had ultra‐micropores with high permeability, gaseous transport, and selectivity when compared with PIM‐1 [143]. The hydrolysis of nitrile groups in PIM‐1 under acidic conditions using pure methane sulfonic acid (MSA) and MSA/water mixtures with systematic variation of reaction parameters gave nitrile functionalized PIM‐1 (nPIM‐1). The transformation of nPIM‐1 through the carboxylic mechanism gave rise to carboxyl‐functionalized PIM‐1 (cPIM‐1). Both nPIM‐1 and cPIM‐1 revealed contraction in interchain spacing due to hydrogen bonding [144]. The combination of cPIM‐1 with sulfonated graphene oxide gave rise to sulfonated graphene oxide‐modified PIM‐1 (SGO‐PIM‐1) with enhanced structural stability, hydrophilicity, and ion exchange capacity in comparison with cPIM‐1 for ion transport and conductivity [145]. The grafting of PIM with thioamide formed thioamide‐modified PIM (tPIM‐1), which was highly microporous, soluble in solvents, and selective for Cd^2+^ and Ag^+^ [146].

## 3 Adsorption Fundamentals

**FIGURE 6:**
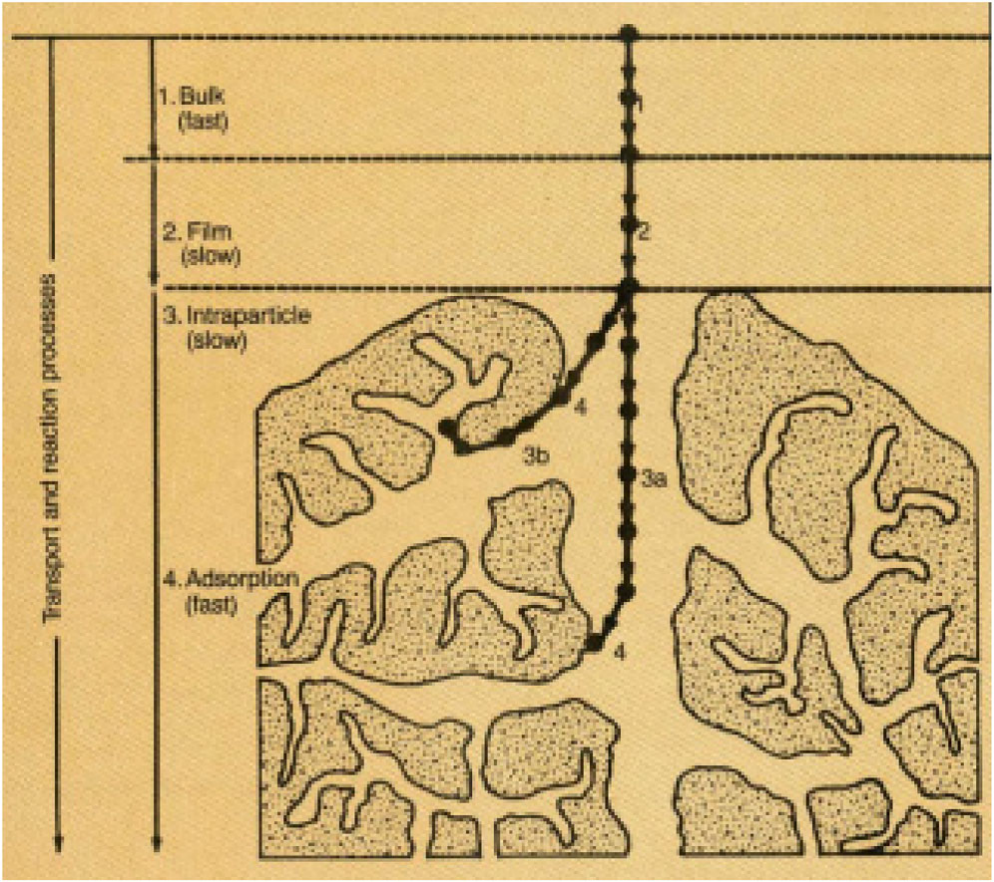
Diffusion processes for adsorbate–adsorbent interactions in microporous adsorbents [157] (Reproduced with permissions: Copyright 1987, American Chemical Society).

Adsorption is the binding or attachment of molecule(s), atom(s), or ion(s) from a solute, liquid, or gas onto a surface. It involves the concentration or aggregation of material(s) (known as adsorbate(s)) in a liquid or gaseous state onto solid materials surface (known as the adsorbent(s)). This phenomenon is greatly influenced by the surface microstructure, surface chemistry, pore size, pore size distribution, and surface area of adsorbent(s). The versatility, benignity, and facile‐tuning of the adsorption technique place it at a vantage position over other separation techniques utilized for the removal of organic contaminants in water and wastewater contaminated. Adsorption is a unique separation technique due to the fact that it takes into cognizance the aggregation or accumulation of more than one adsorbate on the surface of a solid. This technique has garnered a wide array of global interest and application in the treatment of water and wastewater polluted with organics and other contaminants. Rudimentarily, the global application of adsorption for the treatment of water and wastewater underscores the fact that it has been found to be more efficient and effective for the treatment, disinfection, purification, and remediation of water and wastewater over the last two decades. Water treatment technology hinged on adsorption is a promising sustainable technology that contributes to the provision of potable water and sanitation for all by 2030, which is the sixth sustainable development goal (SDG‐6). The adsorption process actively involves the disinfection of pathogens (deactivation and neutralization of pathogens) and the removal of organic and inorganic contaminants (treatment and purification of water containing toxic chemicals). Cogently, adsorbents are porous materials with varying degrees of surface area and pore size that can be tuned and engineered to high selectivity and specificity for target molecules or ions (hydrophobic or hydrophilic) [147, 148, 149, 150, 151–152]. The surface chemistry of various adsorbents is tailored toward the affinity of adsorbents’ surface for the target molecules or ions, ionic radius and structure of molecules or ions, ionic strength, solubility and speciation of ions, and equilibria/mechanistic parameters (isotherms, kinetics, thermodynamics, mass transfer, diffusion processes, etc.) for different adsorption systems. Researchers have proposed the occurrence of adsorption in three consecutive steps, which are; (a) film or boundary layer diffusion‐the steady migration of molecules or ions from the bulk solution to the external surface or boundary layer, (b) intraparticle diffusion‐steady migration of molecules or ions from the external surface or boundary layer to the intraparticles (probably the rate determining step of the adsorption process) and (c) pore or internal surface diffusion diffusion‐steady migration of molecules or ions from the intraparticles to the pores of the adsorbents [153, 154–156] (Figure 6).

## 4 Mechanisms of Adsorption

**FIGURE 7:**
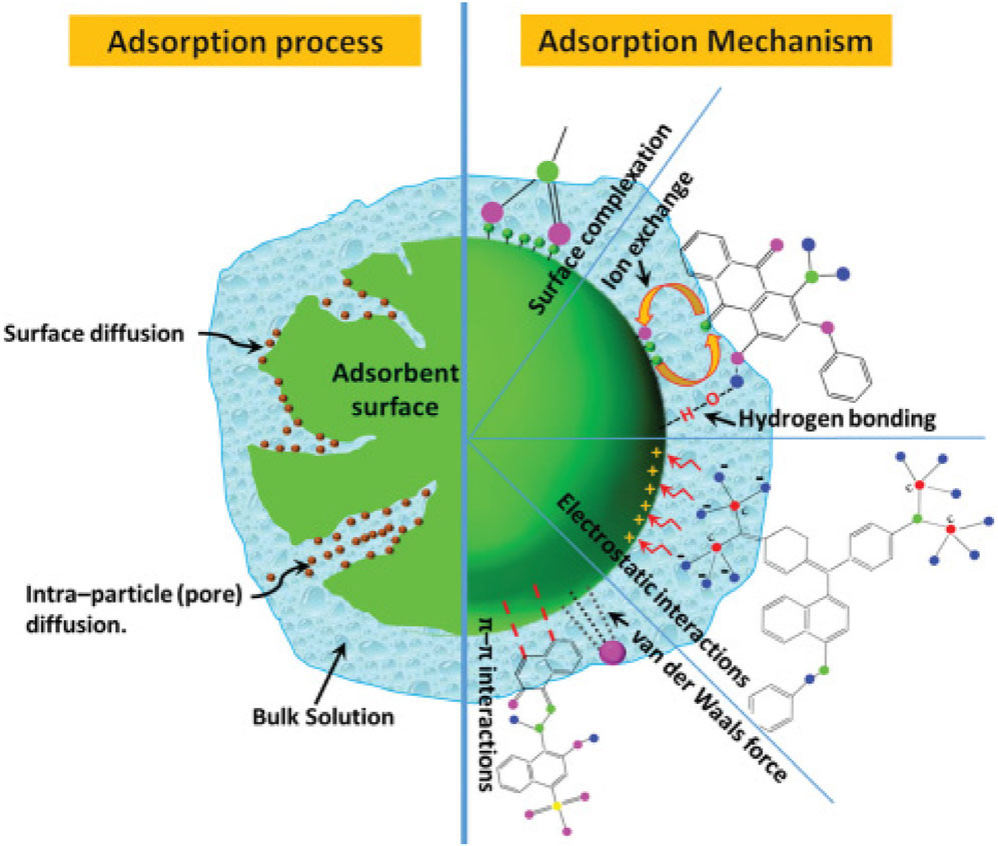
The process and mechanism of the adsorption from bulk aqueous medium [169] (Reproduced with permissions: Copyright 2021, Royal Society of Chemistry).

Adsorption processes are essentially controlled by two mechanisms, which are physical interaction (physisorption) and chemical interaction (chemisorption). Physisorption involves the interplay of some weak intermolecular forces such as weak van der Waal forces (ion–ion interactions, dipole–dipole interactions, hydrogen bonding, and London dispersion forces) between target molecules or ions (adsorbates) and adsorbents’ surfaces. Conversely, chemisorption involves the formation of strong chemical bonds between electron donors and receptors. These electron donors and receptors play vital roles in the chemical interaction that leads to the formation of bonds. The nature of chemical bonds formed during chemisorption may be ionic or covalent. The mechanisms of adsorption processes are observed in adsorbate–adsorbent interactions, which involve *π–* *π* stacking, ion–ion interactions, dipole–dipole interactions, hydrogen bonding and London dispersion forces, ion exchange, complexation, electrostatic interaction, microprecipitation, ligand exchange, chelation, etc. [158, 159, 160, 161–162] (Figure 7).

It is interesting to note that chemisorption produces stronger and more selective and specific bonds than physisorption. The intermolecular forces involved in physisorption are nonselective and nonspecific. Some adsorption processes are both physisorptive and chemisorptive, based on the physicochemical properties of the surfaces of adsorbents and environmental conditions. In some cases, there is a preponderance of either of the two mechanisms, which leads to increased selectivity and specificity of adsorbents for target molecules or ions. Also, adsorption processes can be hydrophilic or hydrophobic in nature, depending on the adsorbate–adsorbent interactions. When adsorbate–adsorbent interactions are hydrophilic, hydrophilic adsorption takes place, and adsorbate–adsorbent interactions that are hydrophobic result in hydrophobic adsorption [163, 164–165].

Physisorption and chemisorption are observed as reversible and nonreversible reactions in adsorption systems at high pressures and elevated temperatures, respectively. Proper understanding of these mechanisms is salient for the engineering of process optimization, process design, adsorbent efficacy, and adsorbent efficiency for adsorption systems. This is highly driven toward exploiting the unique merits and eco‐sustainability of adsorption for the treatment of water and wastewater laden with organic contaminants [166, 167–168].

## 5 PIMs: Adsorbents‐of‐the‐Future

In recent times, PIMs have risen to be promising adsorbents for water purification exceptional characteristics and features, which include engineered surface chemistry, surface texture and morphology, tunable selectivity, tailored specificity to target molecules or ions, high permeability, tunable thermal and mechanical stability, high‐level chemical resistance, among others. They possess excellent surface area and pore structure, and well‐arrayed pore size distribution when compared to other porous adsorbents. These textural properties and surface chemistry of PIMs ultimately facilitate the interaction of target molecules or ions to their active sites via a fast diffusion process. The versatility, adaptability, and scalability of PIMs make them adsorbents‐of‐the‐future for the treatment of point‐of‐use water for domestic purposes and purification of wastewater for large‐scale applications. The applications of PIMs for the improvement of water quality are geared to be sustainable strategies to proffer solutions to the global potable water crisis. The re‐engineering of PIMs with respect to their surface chemistry, morphology, and texture will enhance their multifarious ability for applications as innovative and next‐generation adsorbents for sustainable water treatment, water recycling and recovery, global best practice in sustainable water management and preservation of water resources for a clean and safe environment [170, 171, 172, 173, 174, 175, 176, 177, 178, 179, 180, 181, 182–183]. Sporadically, PIMs are used for the adsorption of ions or molecules due to their special structure and intrinsic microporosity. Their excellent hydrophobicity and high selectivity are based on their interactions with desired target ions or molecules. The applications of PIMs for the molecular separation of organic contaminants are attracting the attention of Researchers due to their unique post‐synthetic tunability, interconnected pores, and monomer engineering design. These are observed in the large unstacked aromatic content of rigidly built blocks, contortion sites, aromatic expanses, constant structural cavities, and inefficient solid state packing, which results in excellent microporosity [104, 184, 185, 186, 187, 188, 189–190]. The alarming rate at which the global demand for better water quality has increased the quest for advanced adsorbents for the treatment of organically contaminated water [184, 190, 191].

## 6 The Applications of PIMs for the Removal of PCs and Organic Dyes in Water

The quest for the supply and demand of potable water is alarmingly increasing all over the world today. For this reason, the safety of water is a salient global issue that cannot be overemphasized. Water contamination has risen to be a major environmental challenge worldwide. The United Nations estimated that around 3.1% of deaths worldwide, which is over 1.7 million deaths annually caused by unsafe or inadequate access to water [155, 192, 193]. To date, there is restricted access to potable water in all the continents of the world, due to growing industrialization. Water is essential for multifarious activities, which include household usage, industrial manufacturing, hospitals/clinics, power, agriculture, food processing and packaging, etc. These processes generate a mammoth amount of wastewater that constitutes serious problems and a menace to the continuous existence of the environment and ecosystem. However, the presence of limited freshwater has grossly decreased the quality and quantity of aquatic life in general. This has a tremendous negative impact on the availability of global freshwater. The purification/treatment and recycling of wastewater to give potable water are auspicious technologies that will end its shortage in the world. Also, access to safe drinking water is not only a human right but also a necessary factor for economic productivity and technological development. The need for the global development of efficient and affordable technologies to improve the quality of water to meet human and environmental needs is increasing daily, and this cannot be overemphasized. Therefore, it has become imperative to proffer the panacea or solution to these life threats [193, 194–195]. The contamination of water sources surface water and groundwater) with PCs and has raised cogent questions with respect to human health, ecology, and economic impacts. Most frequently, surface water and groundwater contaminations are evident from influents and effluents of wastewater treatment plants (WWTPs).

Also, in recent times, PCs and organic dyes have been found in surface water and groundwater around the world. These water contaminants are deleterious to humans, flora, and fauna. PCs have been at the forefront of improved quality of health for some decades due to the fact that they have helped to cure diseases, accelerate health wellbeing, and boost life expectancy. Hence, this medical breakthrough has led to their swift release into water bodies. In 2013, the United States Food and Drug Administration (US FDA) approved more than 100 PCs for medical applications. Due to the nonvolatility and relatively high polarity of PCs, they are typically found in ng.L^−1^ to µg.L^−1^ in the environment. Also, the frequent production and consumption of PCs has increasingly led to their release into the water bodies and consequent in their fate, virulence, and contingency in the aquatic ecosystem. Hence, they are found in varying concentrations in surface water, groundwater, and WWTPs [196, 197–198]. The environmental contamination resulting from the huge release of PCs into surface water, groundwater, and WWTPs is a growing concern in the world today. Globally, >500 PCs have been detected and identified in wastewater. PCs are chemical compounds that are deleterious to human lives and the environment at large at certain doses. Minuscule concentrations (0.6–76 ng.L^−1^) of PCs have a great adverse impact on the environment and on human health [81, 194, 196, 199, 200, 201–202].

On the other hand, organic dyes are the most viable sources of pollutants in industries. They are discharged from food, agricultural, wood, leather, paper and pulp, textile, paints, lacquer and varnishes, adhesives, cosmetics, and PCs industries. The disposal of organic dyes into the aquatic environment is precarious to human health and aquatic lives due to their ability to infertility, deoxyribonucleic acid dysfunction, asthma, allergies, dermatitis, dizziness, irritability, diarrhea, nausea blood, vomiting, leukamia (blood cancer), bladder cancer, bone marrow cancer, hyperactivity, jaundice, mental illness, cyanosis and angioderma [169, 203, 204–205].

Researchers have successfully utilized PIMs for the adsorption of PCs according to the literature in recent years, as shown in Table 1. Al‐Hetlani et al. [206] applied hydrocarbon‐based PIMs (TPB‐HC and TRIP‐HC) and sulfonic acid‐based PIMs (TPB‐SO_3_H and TRIP‐SO_3_H) for the adsorption of three tricyclic antidepressants, desipramine (DES), nortriptyline (NOR), and imipramine (IMI) from contaminated water. The adsorption capacities of these PIMs, TPB‐HC, TRIP‐HC, TPB‐SO_3_H, and TRIP‐SO_3_H for DES, NOR, and IMI are 136.90, 144.90, and 175.40 mg g^−1^; 133.30, 153.90, and 166.70 mg g^−1^; 384.60, 303.00, 312.50 mg g^−1^ and 312.50, 196.00, and 294.10 mg g^−1^ respectively. Also, Al‐Hetlani and her colleagues applied TPB‐HC, TRIP‐HC, TPB‐SO_3_H, and TRIP‐SO_3_H for the adsorption of DES, NOR, and IMI in wastewater. Their results indicated TPB‐HC, TRIP‐HC, TPB‐SO_3_H, and TRIP‐SO_3_H adsorbed 0.51, 0.55, and 0.63 mmol.g^–1^; 0.50, 0.58, and 0.59 mmol.g^–1^; 1.44, 0.81 and 1.11 mmol.g^–1^ and 1.17, 0.74 and 1.05 mmol.g^–1^ respectively [206]. Alnajrani and Alsager [207] applied PIM‐1 for the adsorption of doxycycline, ciprofloxacin, penicillin G, and amoxicillin. Their research findings revealed that PIM‐1 was removed at mg g^−1^, respectively.

**TABLE 1:** The removal of PCs and organic dyes by different PIMs.

PIMs	PCs/organic dyes	*q*_*e*_ (mg.g^−1^)	References
TPB‐HC	DES	136.90	[206]
	NOR	144.90	[206]
	IMI	175.40	[206]
TRIP‐HC	DES	133.30	[206]
	NOR	153.90	[206]
	IMI	166.70	[206]
TPB‐SO_3_H	DES	384.60	[206]
	NOR	303.00	[206]
	IMI	312.50	[206]
TRIP‐SO_3_H	DES	312.50	[206]
	NOR	196.00	[206]
	IMI	294.00	[206]
PIM‐1	Doxycycline	189.00	[207]
	Ciprofloxacin	33.10	[207]
	Penicillin G	257.00	[207]
	Amoxicillin	213.00	[207]
TPB‐SO_3_H	MG	303.03	[83]
	MO	109.89	[83]
TRIP‐HC	MG	294.12	[83]
	MO	270.27	[83]
TRIP‐NH_2_	MG	113.64	[83]
	MO	75.76	[83]
TRIP‐NO_2_	MG	161.29	[83]
	MO	30.39	[83]
Borane Dimethyl Sulfide modified PIM‐1	MO	312.50	[208]
MePIM‐SBF	MB	84.00	[206]
tBuPIM‐SBF	MB	101.00	[206]
AO‐PIM‐1	MB	81.30	[209]
	MO	86.70	[209]
Dense film PIM‐1	SB‐35	41.63	[210]
	oRO	65.72	[210]
Electrospun PIM‐1	SB‐35	42.34	[210]
	oRO	66.89	[210]
11% carboxylated PIM‐1	MB	22.00	[211]
37% carboxylated PIM‐1	MB	51.00	[211]
51% carboxylated PIM‐1	MB	183.00	[211]
Hydrolyzed PIM‐1/Fe_3_O_4_	MB	413.20	[213]
AOPIM‐1/alginate beads	MG	1,023.00	[212]
sodium alginate/AO‐PIM‐1	RhB	1,648.30	[142]
hydrolyzed PIMs microfibers	MB	424.80	[214]
	SafO	364.29	[214]
	MV	317.26	[214]
PIM‐1 microparticles	MG	46.00	[215]

Amin et al. [83] reported that TPB‐SO_3_H, TRIP‐HC, amino‐based PIM (TRIP‐NH_2_) and nitro‐based (TRIP‐NO_2_) PIM had uptake capacities for MG and MO as 303.03 mg g^−1^ and 109.89 mg g^−1^, 294.12 mg g^−1^ and 270.27 mg.g^−1^, 113.64 mg g^−1^ and 75.76 mg g^−1^ and 161.29 mg g^−1^ and 30.39 mg g^−1^ respectively. Satilmis and Uyar modified PIM‐1 with borane dimethyl sulfide, which showed a high adsorption capacity for methyl orange (MO), which was 312.50 mg g^−1^ [208]. Al‐Hetlani and her Colleagues applied alkyl spirobifluorenes (SBF)‐based PIMs, MePIM‐SBF and tBuPIM‐SBF for the adsorption of methylene blue (MB) from wastewater, which gave 84.00 mg g^−1^ and 101.00 mg g^−1^ of respectively [206]. Satilmis applied amidoxime‐modified PIM‐1 (AO‐PIM‐1) for the concurrent adsorption of cationic and anionic organic dyes at low pH. AO‐PIM‐1 removed 81.30 mg g^−1^ and 86.70 mg g^−1^ of MB and MO, respectively, at pH 6.0 and 25°C [209].

Zhang et al. observed that at initial concentrations of 10 and 40 mg.L^–1^, PIM‐1 dense films removed 9.56 and 41.63 mg g^−1^ of solvent blue 35 (SB‐35), then 14.35 and 65.72 mg g^−1^ of oil red O (oRO) from water, respectively. Also, PIM‐1 electrospun fibers removed 10.06 and 42.34 mg g^−1^ of SB‐35 and 14.19 and 66.89 mg g^−1^ of oRO, respectively. In this research, the rates of adsorption of SB‐35 and oRO onto PIM‐1 electrospun fibers were greater than those of PIM‐1 dense films [210].

Satilmis and Budd achieved the uptake of MB by PIM‐1, 11% carboxylated PIM‐1 (AmD‐7), 37% carboxylated PIM‐1 (AcD‐21) and 51% carboxylated PIM‐1 (AcD‐23) at pH values of 3.0, 5.6, 10.0 with adsorption capacities of 22.00, 51.00, 53.00 mg g^−1^; 51.00, 175.00, 210.00, 183.00, 285, 350 mg g^−1^ respectively [211].

Also, for safranin O (SafO), its uptake by AmD‐7, AcD‐21 and AcD‐23 at pH values of 3.0, 5.6, 10.0 with adsorption capacities of 34.00, 100.00, 154.00 mg g^−1^; 25.00, 215.00, 297.00, 158.00, 467.00, 529.00 mg g^−1^ respectively. Adsorption capacities of MB and SafO increased with increasing pH values.

**FIGURE 8:**
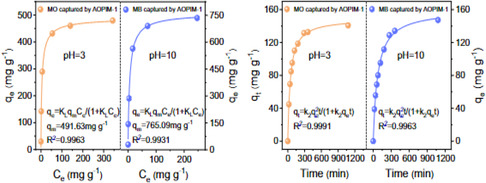
The adsorption of MO and MB by AOPIM‐1 at acidic and basic conditions, respectively [27].

For Amaranth (Amth), its uptake by ethanolamine modified PIM‐1 (EaP‐6) and amine PIM‐1 (aM‐10) at pH values of 3.0, 4.5, 10.0, with adsorption capacities of 636.00, 325.00, 212.00 and 744.00, 135.00, and 140.00 mg g^−1^ respectively. Also, for acid red (AcR), its uptake by EaP‐6 and aM‐10 at pH values of 3.0, 4.5, 10.0, with adsorption capacities of 968, 525.00, 334.00, and 1240.00 mg g^−1^, 393.00, 294.00 mg g^−1^, respectively. For orange II (OrII), its uptake by EaP‐6 and aM‐10 at pH values of 3.0, 5.6, 10.0, with adsorption capacities of 754.00, 425.00, 402.00 and 1093.00, 275.00, and 195.00 mg g^−1^ respectively. Adsorption capacities of MB and SafO decreased with increasing pH values. Satilmis and his Colleagues observed that PIM‐1 synthesized at 65°C (SPIM) adsorbed 3.00 mg g^−1^ and 6.00 mg g^−1^ of OrII and SafO, respectively, which are organic dyes. Also, 3–24 h‐ethanolamine modified SPIM at 120°C adsorbed 91.00–363.00 mg g^−1^ and 6.00–15.00 mg g^−1^ of OrII and SafO, respectively. In a similar correlation, 6–52 h‐ethanolamine modified TPIM (PIM‐1 synthesized at 160°C) at 90°C and 105°C adsorbed 99.00–545.00 mg g^−1^ and 1.00–7.00 mg g^−1^ of OrII and SafO, respectively. They further reported the adsorption capacities of 9–90 h diethanolamine modified TPIM at 120°C and 150°C adsorbed 6.00–287.00 mg g^−1^ of OrII, respectively. Xu et al. [212]. reported that AOPIM‐1/alginate beads adsorbed 1023.00 mg g^−1^ of MG, and Wang et al. [27]. reported the adsorption of MO and MB by AOPIM‐1, whose uptake capacities for MO and MB are 491.63 mg g^−1^ and 756.09 mg g^−1^, respectively (Figure 8).

Hydrolyzed PIM‐1/Fe_3_O_4_ composite adsorbed 413.2 mg g^−1^ of MB according to the report of Zhang and his Colleagues [213]. Also, Yang et al. [142]. reported that sodium alginate/AO‐PIM‐1 removed 1,648.30 mg g^−1^ of RhB. Zhang et al. [214]. utilized hydrolyzed PIMs microfibers for the adsorption of MB, SafO, and methyl violet (MV), with adsorption capacities of 424.80, 364.29 and 317.26 mg g^−1^ respectively. Also, Lu et al. [215]. utilized PIM‐1 microparticles with an adsorption of 46.00 mg g^−1^ for MG.

## 7 Future Outlook and Conclusion

The environmental applications of PIMs are an emerging research interest that explores the excellent surface properties of PIMs in terms of their high surface areas and large pore sizes, which could be engineered and tuned, so as to increase their free volume, microporosity, permeability, exceptional chain rigidity, and excellent solubility. The review explores the recent advances in the applications of PIMs for the sequestration/removal of some organic contaminants (PCs and organic dyes). This review paper is a scrupulous report of a cocktail of published literature on the various utilizations of PIMs and their modified analogs for the removal of PCs and organic dyes. It is noteworthy to state here that the future perspectives of PIMs for the adsorption of PCs and organic dyes should be directed to the following;

i. The free volume, permeability, and selectivity of PIMs, especially when improved, have aroused the enthusiasm of Researchers globally to apply them for pervaporation, membrane separation, and catalysis extensively. These are shown in myriads of papers published on their applications for gases and volatile components separation, desalination, reduction of gases, etc. Future perspectives on the application of PIMs for the adsorption of PCs and organic dyes should be focused on their engineered modifications, tailored to increase the microporosity, high selectivity, high thermal stability, high molecular diffusivity, and reduced solubility. This will improve their properties for adsorptive applications of PCs and organic dyes.
ii. There is a need to develop cutting‐edge research strategies to modify PIMs with other porous organic polymers, such as porous crosslinked polymers, conjugated microporous polymers, and HCPs, so as to understand and ascertain the impact of their synergism on the adsorption capacities of PIMs for PCs and organic dyes. Similarly, the synergistic effects of other porous materials, such as zeolites, porous carbons, MOFs, covalent organic frameworks, porous aromatic frameworks, and covalent triazine frameworks, when used to modify PIMs, are worthy to be study in the near future. This will establish the impact of these modifiers on the adsorption capacities of PIMs for PCs and organic dyes.
iii. Modifications of PIMs should be geared toward improving their hydrophilicity with cutting‐edge materials such as MXenes (surface‐active hydrophilic nucleophiles) and hydrophobicity (using nonpolar organic molecules), so as to increase their inherent selectivity for PCs, organic dyes, phthalates, toxic metals, polyfluoroalkyl substances, polyaromatic hydrocarbons, endocrine disrupting chemicals, microplastics, etc.

## Author Contributions

**Martins O. Omorogie**: conceptualized research, curated data, analyzed data, wrote and edited the manuscript, obtained and administered research funds.

## Conflicts of Interest

The author declare no conflicts of interest.
